# Biophenolic Profile Modulations in Olive Tissues as Affected by Manganese Nutrition

**DOI:** 10.3390/plants10081724

**Published:** 2021-08-20

**Authors:** Nikolina Vidović, Igor Pasković, Igor Lukić, Paula Žurga, Valerija Majetić Germek, Kristina Grozić, Marin Cukrov, Šime Marcelić, Dean Ban, Nassima Talhaoui, Igor Palčić, Vedran Rubinić, Smiljana Goreta Ban

**Affiliations:** 1 Department of Agriculture and Nutrition, Institute of Agriculture and Tourism, K. Huguesa 8, 52440 Poreč, Croatia; nikolina@iptpo.hr (N.V.); igor@iptpo.hr (I.L.); grozic@iptpo.hr (K.G.); mcukrov@iptpo.hr (M.C.); dean@iptpo.hr (D.B.); palcic@iptpo.hr (I.P.); smilja@iptpo.hr (S.G.B.); 2 Centre of Excellence for Biodiversity and Molecular Plant Breeding, Svetošimunska 25, 10000 Zagreb, Croatia; 3 Teaching Institute of Public Health Primorsko-Goranska County, Krešimirova 52a, 51000 Rijeka, Croatia; paula.zurga@zzjzpgz.hr; 4 Department of Food Technology and Control, Faculty of Medicine, University of Rijeka, Brace Branchetta 20, 51000 Rijeka, Croatia; valerija.majetic@medri.uniri.hr; 5 Department for Ecology, Agronomy and Aquaculture, University of Zadar, Trg kneza Višeslava 9, 23000 Zadar, Croatia; simemarcelic@unizd.hr; 6 Highfield Campus, University of Southampton, Southampton SO17 1BJ, UK; nassima.talhaoui@gmail.com; 7 Department of Soil Science, Faculty of Agriculture, University of Zagreb, Svetošimunska 25, 10000 Zagreb, Croatia; vrubinic@agr.hr

**Keywords:** *Olea europaea* L., root, stem, leaves, cv., ‘Istarska bjelica’

## Abstract

Manganese (Mn) is an essential element that intervenes in several plant metabolic processes. The olive tree, and its fruits and leaves, are known as a source of nutraceuticals since they are rich in biophenols. However, there is still a serious lack of data about biophenolic distribution in olive stems and roots under Mn fertilisation. In this context, our study aimed to examine the effects of Mn fertilisation on the biophenolic profile in the leaves, stems, and roots of the ‘Istarska bjelica’ olive cultivar. The experiment was set up in a greenhouse, during a period of five months, as a random block design consisting of three treatments with varying Mn concentrations in full-strength Hoagland’s nutrient solution (0.2 µM Mn, 12 µM Mn, and 24 µM Mn). The obtained results indicate that the amount of Mn in the examined olive plant tissues was significantly higher under 12 µM Mn and 24 µM Mn treatments compared to that of the 0.2 µM Mn treatment. While the concentration of biophenols varied in roots depending on the compound in question, a strong positive impact of the increased Mn concentration in nutrient solution (12 µM Mn and 24 µM Mn) on the concentrations of the main biophenolic compounds was observed in stems. The concentration of oleuropein in leaves almost doubled at 24 µM Mn, with the highest Mn concentration, as compared to the 0.2 µM Mn treatment. The obtained results led to the conclusion that the supply of Mn could enhance the concentration of some biologically active compounds in olives grown hydroponically, implying a critical need for further investigation of Mn fertilisation practices in the conventional olive farming system.

## 1. Introduction

The olive tree (*Olea europaea* L.) is one of the oldest and emblematic cultivated trees in the Mediterranean, traditionally grown in the coastal areas of southeastern Europe and northern Africa. Several studies demonstrated that olive oil consumption procures multiple advantages to humans at different levels due to its nutritional and health care benefits [1]. Consequently, the cultivation of olive trees spread globally during the past three decades [2], leading to the expanded production of waste or residues derived from olive tree cultivation and the olive processing industry.

Olive leaves are considered one of the most significant by-products in olive oil production [3], whose accumulation may surpass one million tons per year [4]. Although there are many potential ways to use olive leaves, only a small fraction are actually exploited, and the vast amount of this residual material is usually burned or otherwise discarded [5]. Since it is well known that biophenolic compounds from olive leaf extracts may exert beneficial properties for human health, a large amount of bioactive phenols is consequently squandered in this way. According to the literature, the pharmacological properties of biophenols include antioxidant [6], anti-inflammatory [7,8], antimicrobial [9], antiviral [10] and anti-carcinogenic activities [11], as well as beneficial cardiovascular effects [12]. These effects are attributed strictly to the presence of a range of compounds primarily dominated by phenolic acids (ferulic, vanillic, and caffeic acid), phenolic alcohols (tyrosol and hydroxytyrosol), flavonoids (luteolin-7-*O*-glucoside, apigenin-7-*O*-glucoside, rutin, luteolin-4′-*O*-glucoside, apigenin, and luteolin), hydroxycinnamic acid derivatives (verbascoside), and secoiridoids (oleuropein) [3,11,13,14,15]. In addition to leaves, pruning generates a significant amount of olive stems, an underestimated source of biophenols [16]. When compared to leaf extracts, biophenolic compounds derived from stems were never considered valuable in folk or modern medicine [17]. However, recent studies demonstrated that olive stems yield significant amounts of secoiridoid compounds such as oleuropein [18]. These findings make stems a promising raw material to be considered in the green and circular economy system as a potential candidate for the extraction of biophenols.

Biophenolic compounds mostly derive from the shikimic acid biosynthetic pathway in which phenylpropanoids are formed. The first reaction is catalyzed by 3-deoxy-D-arabino-heptulosonate-7-phosphate synthase (DAHPS), an enzyme that requires Mn^2+^ for its activity [19]. Additionally, Mn ions are found to be linked to the functioning of phenylalanine ammonia-lyase (PAL). PAL is the enzyme responsible for the initiation of the phenylpropanoid pathway and the subsequent increase in the concentration of biophenols [20]. Several authors investigated the effect of Mn nutrition on biophenolic content in different species in order to support the above claim. Brown et al. [21] reported that Mn deficiency decreased biophenols in wheat shoots. Similarly, the total phenolic content (TPC) in tomato fruit increased when the plant was exposed to higher Mn concentrations [22]. Increased concentrations of flavonoids in water mint were linked to higher Mn concentration in nutrient solution [23]. According to Farzadfar et al. [24], the levels of individual biophenolics in feverfew plants were highly dependent on Mn supply, and the increment in their concentration was proportional to the amount of Mn applied. Additionally, in our previous study [25], foliar Mn application caused a significant increase in levels of the most prominent olive phenolic compound, oleuropein. Whether that implies a certain Mn intervention in the putative secoiridoid pathway, or its role in some other unexplained chain of metabolic events leading to the latter effect, remains unknown to date. In addition, the data on the resulting variation of other biophenolic compounds in olive tissues are still scarce [16,25,26]. Seeing the importance of this micronutrient in the synthesis of biophenols, further investigation of the effects of Mn on the biophenolic content in olive leaves is needed. Moreover, the impact of Mn nutrition on the biophenolic profiles of olive stems and roots and on their long-distance leaf to root transport is also still unknown and needs to be studied.

This work aimed to determine the effect of controlled Mn root fertilisation on the phytochemical composition of olive leaves, stems, and roots of ‘Istarska bjelica’ olive cultivar (cv.) grown hydroponically during five months. Considering the importance of biophenolic compounds, the obtained results could contribute to the development of targeted fertilisation programs.

## 2. Results

### 2.1. Concentration of Mn and Biophenols in Olive Leaves, Stems, and Roots

**Figure 1:**
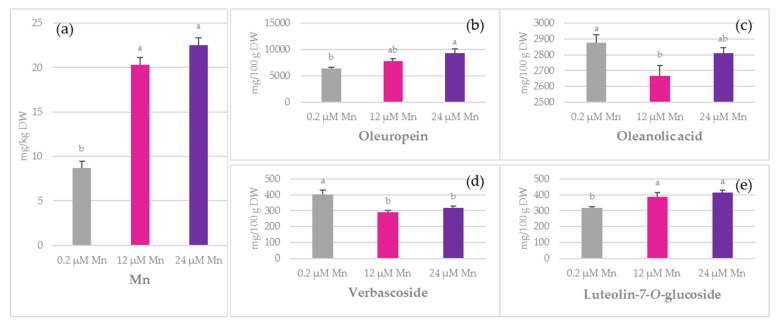
The effect of manganese (Mn) application on: (**a**) Mn, (**b**) oleuropein, (**c**) oleanolic acid, (**d**) verbascoside, and (**e**) luteolin-7-*O*-glucoside concentrations in olive leaves of ‘Istarska bjelica’ olive cv. (mean values ± SE, *n* = 4). Mean values followed by different letters are significantly different at *p* < 0.05 according to Tukey’s test. DW—dry weight.

The concentration of Mn and the most common biophenols in the olive leaves of ‘Istarska bjelica’, cultivated hydroponically and treated with three different Mn concentrations (0.2 µM Mn, 12 µM Mn, and 24 µM Mn), are reported in Figure 1. 

A significant increase in Mn concentration was observed in leaves, ranging from 8.70 mg/kg dry weight (DW) at 0.2 µM Mn to 22.52 mg/kg DW at 24 µM Mn. The major bioactive compounds found in leaves were oleuropein (6354–9313 mg/100 g DW) and oleanolic acid (2877–2809 mg/100 g DW), followed by verbascoside (403–291 mg/100 g DW) and luteolin-7-*O*-glucoside (318–416 mg/100 g DW) (Figure 1). The increase in Mn concentration in nutrient solution positively impacted the concentration of oleuropein, luteolin-7-*O*-glucoside, verbascoside, oleanolic acid, luteolin, and apigenin in leaves (Figure 1, Table S1). The concentration of verbascoside was higher at 0.2 µM Mn than at 12 and 24 µM Mn. Oleuropein and luteolin-7-*O*-glucoside, on the other hand, showed an increase in their concentrations that was proportional to the increment in Mn concentration (Figure 1). The amount of apigenin and luteolin was the lowest in the treatment with the highest Mn concentration (24 µM Mn). A negative effect of Mn amendment on the oleanolic acid concentration was observed at 12 µM Mn as compared to the 0.2 µM Mn treatment. In contrast, the concentration of Mn in nutrient solution did not significantly affect the concentrations of hydroxytyrosol, tyrosol, vanillin, vanillic acid, catechin, rutin, and apigenin-7-*O*-glucoside in leaves (Table S1). The total phenolic content (TPC) in leaves remained unchanged under the applied treatments. 

**Figure 2:**
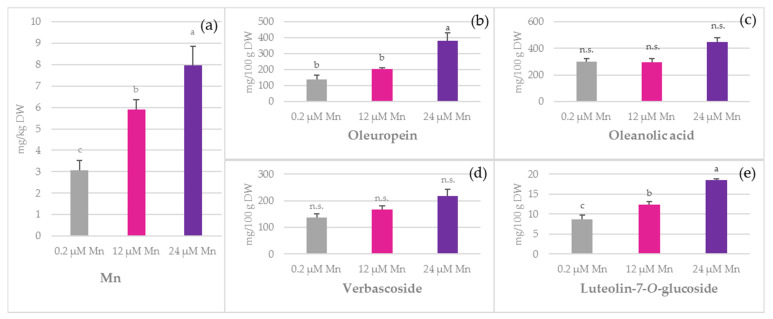
The effect of manganese (Mn) application on: (**a**) Mn, (**b**) oleuropein, (**c**) oleanolic acid, (**d**) verbascoside, and (**e**) luteolin-7-*O*-glucoside concentrations in olive stems of ‘Istarska bjelica’ olive cv. (mean values ± SE, *n* = 4). Mean values followed by different letters are significantly different at *p* < 0.05 according to Tukey’s test. DW—dry weight.

In stem samples, the concentration of Mn increased from 3.06 mg/kg DW under 0.2 µM Mn treatment to 7.97 mg/kg DW under 24 µM Mn treatment (Figure 2). The most abundant bioactive compounds in stems were oleanolic acid, oleuropein, and verbascoside, while the highest concentration among flavonoids was obtained for luteolin-7-*O*-glucoside (Figure 2). 

A significant increase in oleuropein, luteolin-7-*O*-glucoside, vanillin, rutin, hydroxytyrosol, apigenin, vanillic, and caffeic acid concentration was observed (Figure 2, Table S2). Among the investigated simple biophenols, concentrations of hydroxytyrosol and vanillin were higher at 24 µM Mn when compared to the other two treatments (Table S2). Vanillic and caffeic acid concentrations in leaves were higher at 24 µM Mn as compared to the 0.2 µM Mn treatment (Table S2). The total amount of flavonoids showed a significant increase at 24 µM Mn with respect to the 0.2 µM Mn treatment (data not shown). Among all the investigated flavonoids, only the concentration of luteolin did not vary. The most pronounced change was noticed for luteolin-7-*O*-glucoside, whose concentration was doubled at 24 µM Mn compared to 0.2 µM Mn (Figure 2). 

The concentration of Mn in nutrient solution significantly affected the stem oleuropein levels in a dose-dependent manner, increasing its concentration in the stems from 139.45 mg/100 g DW at 0.2 µM Mn to 379.93 mg/100 g DW at 24 µM Mn (Figure 2). The concentration of a triterpene, oleanolic acid, was not significantly increased under the Mn treatments. TPC remained unchanged in stems. 

**Figure 3:**
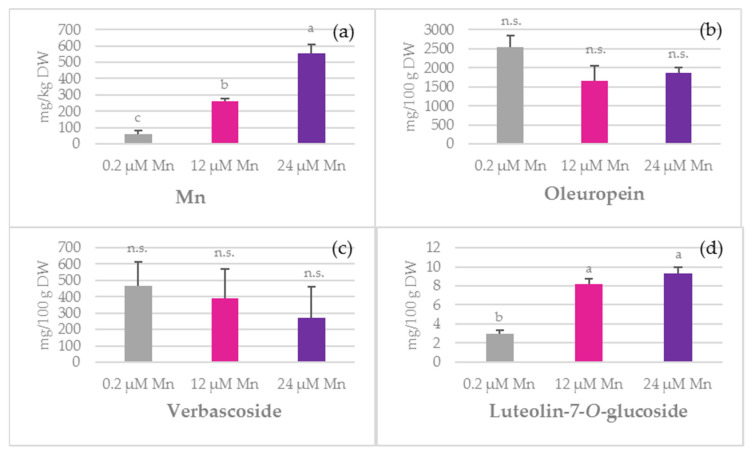
The effect of manganese (Mn) application on: (**a**) Mn, (**b**) oleuropein, (**c**) verbascoside, and (**d**) luteolin-7-*O*-glucoside concentrations in olive roots of ‘Istarska bjelica’ olive cv. (mean values ± SE, *n* = 4). Mean values followed by different letters are significantly different at *p* < 0.05 according to Tukey’s test. DW—dry weight.

The concentration of Mn in the roots significantly increased under the applied treatments. For example, an almost 10-fold increment was recorded for roots at 24 µM Mn (552.99 mg/kg DW) as compared to the 0.2 µM Mn treatment (60.84 mg/kg DW, Figure 3). However, the TPC in roots did not significantly change under the applied treatments. The most widespread biophenols found in roots were oleuropein and verbascoside (Figure 3). Of all the analysed flavonoids, only the presence of apigenin-7-*O*-glucoside and luteolin-7-*O*-glucoside was confirmed, the former being the more abundant one (Table S3 and Figure 3). The concentration of luteolin-7-*O*-glucoside was higher at 12 µM Mn and 24 µM Mn treatments (8.15 mg/100 g DW and 9.31 mg/100 g DW, respectively) as compared to the 0.2 µM Mn treatment (Figure 3). For the apigenin-7-*O*-glucoside concentration, an almost 2.5-fold increment was recorded at 24 µM Mn when compared to the 0.2 µM Mn treatment. In roots, the concentrations of tyrosol and caffeic acid were lower at 12 µM Mn and 24 µM Mn than at 0.2 µM Mn. On the other hand, the presence of oleanolic acid in roots was not detected, while the quantities of the other investigated biophenols did not differ significantly among the treatments (Table S3).

### 2.2. Morphological Parameters

The Mn treatments significantly affected the morphological parameters of ‘Istarska bjelica’ roots, except for the root diameter, where no significant differences were recorded (Table 1). The 12 µM Mn treatment positively affected the total root length, root surface area, and root volume as compared to 0.2 µM Mn, but the further increase in Mn concentration negatively impacted all three parameters (Table 1). None of the Mn-treated plants differed significantly from the non-treated ones with respect to the number of nodes and leaves, plant height, and dry plant tissue biomass (Table S4). 

### 2.3. Total per Plant Quantity of Mn and Biophenols

The results the analysis of TPC, particular biophenols, and Mn were converted to quantities by multiplying the dry weights (DW) of plant tissues with the corresponding concentrations. The total per plant quantities were calculated by summing the corresponding quantities obtained for leaves, stems, and roots (for details, see Section 4).

**Figure 4:**
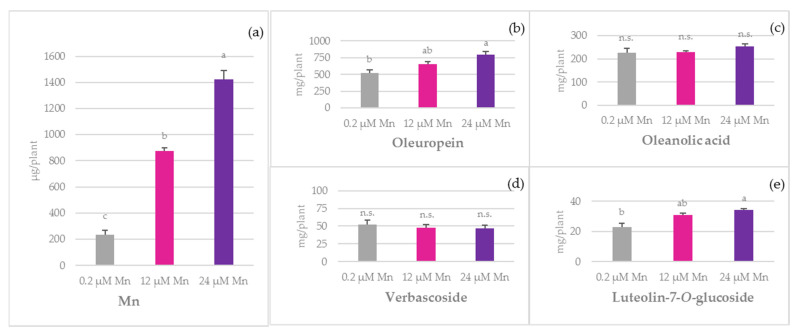
The effect of manganese (Mn) application on the total per plant quantity of (**a**) Mn, (**b**) oleuropein, (**c**) oleanolic acid, (**d**) verbascoside, and (**e**) luteolin-7-*O*-glucoside of ‘Istarska bjelica’ olive cv. (mean values ± SE, *n* = 4). Mean values followed by different letters are significantly different at *p* < 0.05 according to Tukey’s test.

The total per plant Mn quantity increased from 233.5 µg at 0.2 µM Mn to 1423.71 μg at 24 µM Mn, corresponding to a 600% increase (Figure 4). Regarding the total per plant quantity of the investigated bioactive compounds, the most abundant were oleuropein, oleanolic acid, verbascoside, and luteolin-7-*O*-glucoside (Figure 4). The quantities of the other compounds are summarised in Table S5. 

A positive impact of boosted Mn nutrition on the quantities of oleuropein, luteolin-7-*O*-glucoside, and caffeic acid was observed, which increased significantly from 0.2 µM Mn to 24 µM Mn treatment. A significant negative correlation between Mn nutrition and the quantities of particular biophenolic compounds was recorded only in the case of luteolin (*r* = −0.70, *p* = 0.012), while a significant positive correlation was observed for hydroxytyrosol (*r* = 0.61, *p* = 0.036), vanillic acid (*r* = 0.62, *p* = 0.031), caffeic acid (*r* = 0.89, *p* = 0.000), luteolin-7-*O*-glucoside (*r* = 0.81, *p* = 0.001), rutin (*r* = 0.75, *p* = 0.005), and oleuropein (*r* = 0.83, *p* = 0.001) (Table 2). 

### 2.4. Mn Uptake and Use Efficiency

The distribution (%) of the total per plant quantity of Mn in the three vegetative tissues was calculated, and the results are summarised in Table 3. Both of the above-indicated parameters significantly differed among the treatments. When the highest concentration of Mn was applied (24 µM Mn), its percentage in roots increased by 22% as compared to 0.2 µM Mn, consequently decreasing the percentages in stems and leaves. The upward transport of Mn from roots to stems and leaves was expressed as the Mn translocation factor (TF). For leaves, TF ranged from 0.48 at 0.2 µM Mn to 0.15 at 24 µM Mn treatment, respectively. For stems, these values ranged between 0.18 and 0.05 (Table 3), thus confirming the results obtained for Mn distribution. Finally, the Mn use efficiency (MnUE) was further calculated, and the obtained results showed a 6.5-fold increment with the increase in Mn concentration in the nutrient solution (Table 3).

## 3. Discussion

### 3.1. Mn Distribution in Olive Tissues and TF

To the best of our knowledge, no previous studies have been performed to evaluate the effects of Mn nutrition on the amount of biophenols in olive stems and roots. As is well known, a rapid conversion of soluble Mn to insoluble Mn oxides, that are unavailable for plant uptake, occurs in alkaline soils (pH values > 8) [27]. Therefore, in our experiment, the pH of nutrient solutions was maintained below 6.1, with percolate pH varying between 7.1 and 7.8 (data not shown).

Manganese concentration, which can lead to plant deficiency or toxicity symptoms, varies among species and shows the complexity of mechanisms for Mn uptake and its translocation to the shoots, as well as its root radial transport [28,29,30,31,32,33,34,35]. Although our results indicate that the determined Mn leaf concentration can be considered as deficient (<10 mg/kg DW) at 0.2 µM Mn or optimal (>20 mg/kg DW) at 12 µM Mn and 24 µM Mn [36] (Figure 1), there were no differences in dry plant biomass between the treatments (Table S4). 

The functions of Mn in roots are largely unresolved, as are the processes involved in root adaptation to Mn deficiency or excess. Generally, excessive Mn concentrations inhibit root growth by disrupting biosynthesis and transport auxins (hormones), which are essential for plant body development [37]. A reduction in the growth of several plant species that were fertilised with Mn was previously reported [38,39,40,41]. In the present study, the root morphological parameters were significantly affected by the change in the concentration of Mn. The elongation of the total root length, higher root surface area, and root volume was recorded for 12 µM Mn treated plants, but the further increase in Mn concentration caused a significant decrease in the parameters indicated above. This led to the conclusion that optimal Mn concentration for root elongation, surface area, and volume was achieved at 12 µM Mn treatment. According to Chatzistathis [42], for ‘Kothreiki’ and ‘FS-17′ cultivars irrigated with half-strength Hoagland’s nutrient solution, the optimal concentration of Mn was 40 µM.

It was previously published that Mn preferably accumulates in plant shoots than in roots [43,44], but its retention in the roots has been associated with Mn tolerance in some plants [45]. Chatzistathis et al. [46] reported that different Mn distributions are not only a feature of species but also of different genotypes within the same species. Plant species can tolerate excessive Mn concentrations due to their ability to control metal distribution within the plant parts. This implies the accumulation of Mn in some tissues, such as roots that are the first channel of Mn exposure, and low distribution to other tissues, for example, the shoots. Our results confirmed this theory since a large quantity of Mn was retained in the roots (61–83%) compared to that which was translocated in the leaves (28–13%) and stems (11–4%, Table 3). The TF, which is used to measure the efficiency of a plant to translocate Mn from roots to shoots, was decreased from 0.48 to 0.15 in leaves and from 0.18 to 0.05 in stems under the applied treatments, suggesting a good tolerance of ‘Istarska bjelica’ cv. plantlets to higher Mn root concentrations (Table 3). The concentration of Mn in all the examined plant parts, as well as the total per plant Mn quantity, significantly increased with the increase in Mn concentration in the nutrient solution, which confirms previously reported data [40,44]. Expectedly, MnUE significantly decreased with the increase in Mn concentration in nutrient solution (Table 3), suggesting that treated plants did not utilise the whole amount of the available Mn for their biological functions. 

### 3.2. TPC

Biophenols constitute probably the largest group of plant secondary metabolites, showing a diversity of structures, from rather simple, through polyphenols such as flavonoids, to the complex ones, and are widely distributed in plant tissues. While some biophenols are extremely widespread, others are specific for certain plant families or plant organs. In this study, a positive correlation between TPC and Mn concentration was observed in leaves (*r* = 0.75, *p* = 0.005) and stems (*r* = 0.91, *p* = 0.000), as well as between total phenolic quantity (TPQ) and Mn in the whole plant (*r* = 0.65, *p* = 0.022; Table 2). Despite the fact that Mn is associated with an increase in the concentration of biophenols [22,23,24,25], several studies reported possible negative effects of Mn fertilisers on the total amount of biophenols in roots and leaves [43]. In our experiment, the TPC values remained unaltered in all of the plant parts, but were generally higher in leaves compared to the other tissues. Moreover, a significant difference in TPC in olive roots, which can be associated with the increase in Mn root concentration, was not observed between the treatments (Table S3). Similar findings were published by Rengel et al. [47], who suggested low Mn requirements for the biosynthesis of root biophenolics. 

### 3.3. Phenolic Acids

Simple phenolic acids are among the most widely distributed phenolic compounds in plants, occurring as derivatives of either benzoic or cinnamic acid. Despite a rather simple structure, their physiological roles extend from supporting the structural components of the cell walls to the formation of allelochemicals and participation in signalling and defence mechanisms [48]. Moreover, certain phenolic acids take part in the formation of other simple biophenols through a plethora of cascade reactions. For instance, hydroxycinnamic acids, such as caffeic, cinnamic, and ferulic acid, act as precursors of vanillin, each through its own respective pathway [48]. Caffeic acid undergoes two consecutive methylations of its aromatic ring, forming 3,4-dimethoxycinnamic acid, whose methyl group is further hydrolyzed, thus producing vanillic acid that can later be reduced to vanillin [49]. This might explain the higher vanillic to caffeic acid ratio observed in both stems and roots (data not shown), since caffeic acid is consumed for vanillic acid and vanillin synthesis. Both of these acids exhibited similar responses to Mn treatments; however, their trends differed depending on the olive part in question. In stems, elevated Mn supply promoted the concentrations of both caffeic and vanillic acid (Table S2), whereas they decreased in roots (Table S3). Interestingly, of all the investigated plant parts, the levels of vanillic acid were the lowest in leaves where caffeic acid was not even detected. Several studies confirmed that caffeic acid in leaves is often found in minor concentrations, rendering our results in line with those obtained by other authors [3,49,50]. This fact, however, does not overshadow its metabolic significance, since it is a superior radical quencher and antioxidant. Phenolic acids, and in particular caffeic acid, are able to chelate free metals that are involved in radical-generating reactions [49]. Under enhanced Mn supply, a significant decrease in root caffeic acid concentration was recorded. Such an event could indicate metal ion chelation by caffeic acid. Considering the whole plant, both caffeic and vanillic acids correlated significantly with Mn quantity, with correlation coefficients of 0.62 (*p* = 0.031) for vanillic acid and 0.89 (*p* < 0.001) for caffeic acid, respectively (Table 2).

Verbascoside is a slightly more complex molecule synthesized through the esterification of caffeic acid with rutinose and subsequent formation of an ether bond with hydroxytyrosol. It is a valuable compound considered in the treatments of oxidative stress-related diseases [50], and alongside oleuropein, it is the most prominent compound found in roots [51]. In this experiment, its concentration declined in leaves under 12 µM Mn and 24 µM Mn treatments as compared to those under 0.2 µM Mn treatment, while no significant difference was observed for stems and roots (Figure 1d, Figure 2d and Figure 3d). Since it shares the same hydroxytyrosol moiety with oleuropein, the changes in their relationship and ratio might be indicative of certain metabolic adaptations. It is rather interesting how enhanced Mn supply decreased the levels of verbascoside in leaves, while simultaneously increasing oleuropein concentrations. This relationship was also observed in water-stressed olives [51] and during olive pulp maturation [52]. However, these findings were not supported by Ryan et al. [53]. On the basis of our findings, the question arose as to whether Mn could possibly cause the degradation of verbascoside or whether it is a consequence of other physiological events.

### 3.4. Oleuropein

Secoiridoid phenolics are compounds that are derived from a mixed biosynthetic origin (shikimate/mevalonate) where tyrosol or hydroxytyrosol are coupled to an iridoid moiety. Oleuropein is one of the main representatives of secoiridoids [54].

It is important to emphasise that oleuropein plays an important role in scavenging free radicals [53], and its presence may be linked to multiple benefits. For example, it has a role in plant protection against different pathogens, suppresses olive oil deterioration, and prolongs the shelf life of food products. According to Alagna et al. [54], oleuropein is the predominant *ortho*-diphenol that is present in all olive tissues, with the highest distribution in leaves. In this study, the concentration of oleuropein in leaves significantly increased at 24 µM Mn compared to 0.2 µM Mn in nutrient solution that was applied when growing olive plantlets (Figure 1), suggesting a potentially stimulative role of Mn ion on the oleuropein biosynthesis. The initially high oleuropein amount found at 0.2 µM Mn could be ascribed to the type of cultivar since it is known that ‘Istarska bjelica’ is one of the Croatian cultivars with the highest olive leaf oleuropein potential [55]. In addition, the concentration of oleuropein was the highest under 24 µM Mn treatment. Previous reports by Alagna et al. [54] stated that the decline in oleuropein concentration corresponded to an increase in the concentration of such flavonoids as luteolin-7-*O*-glucoside. However, in the current study, such an effect was observed only in roots (Figure 3) and was not observed in other olive tissues. Although oleuropein is found in high concentrations in a large number of *Oleaceae* species, there is still a knowledge gap between its biosynthesis and the targeted Mn fertilisation potential.

### 3.5. Simple Biophenols

In our recent comprehensive study [56] concerning the variation of phenolic and mineral composition in several olive cultivars, a strong positive correlation was found between the concentrations of Mn and tyrosol in leaves (*p* < 0.001, *r* = 0.58). However, the results of this study point to the inability of higher Mn concentrations in nutrient solution to significantly affect the levels of simple biophenols in leaves (Table 2). This could probably be attributed to the high retention of Mn in roots, since the concentration of Mn in leaves in the previous study was 50.31 mg/kg compared to the highest value of 22.52 mg/kg found in this study at 24 µM Mn. It is known that higher Mn concentrations can facilitate the chemical oxidation of certain olive *ortho*-diphenols due to the characteristic catalytic effect of Mn cations [51]. According to Romero et al. [57], specific effects of Mn on certain biophenolic compounds were clarified through observations of their oxidation rates in the presence of Mn cations. Their results showed a gradual increase in the oxidation rate of hydroxytyrosol with the increase in Mn concentrations. Due to the tight relationship between hydroxytyrosol and tyrosol, this could partly explain why root concentration of tyrosol in our trial declined as Mn concentrations increased (Table S3). The concentrations of tyrosol in stems did not significantly change under the applied treatments (Table S2). However, the concentration of hydroxytyrosol in stem samples increased at 24 µM Mn as compared to the 0.2 µM Mn and 12 µM Mn treatments (Table S2). Interestingly, the concentrations of hydroxytyrosol and tyrosol were quite similar in leaves, and hydroxytyrosol dominated in stems, whereas tyrosol was more abundant in roots. Regarding the whole plant, a positive correlation coefficient (*r* = 0.61, *p* = 0.036) between the quantities of Mn and hydroxytyrosol (Table 2) could be an indicator that boosted Mn fertilisation can positively affect the amount of this valuable compound. The concentration of vanillin significantly increased in stem samples in treatments from 0.2 µM Mn to 24 µM Mn (Table S2) and the lowest values were recorded in leaves (Table S1) compared to stems and roots (Table 2 and Table 3).

### 3.6. Oleanolic Acid and Flavonoids

A high amount of pentacyclic triterpenoid oleanolic acid was found in leaves (Figure 1) and stems (Figure 2) in all of the treatments. It is known that Mn is one of the major cofactors of terpene synthase enzymes (TPS) responsible for production of terpene skeletons [58]. The availability of this micronutrient increases the accumulation of monoterpenes in plants [59], while the proportion of sesquiterpenes increases in response to Mn deprivation [60]. The biosynthesis of different terpenes depends on the levels of chemical reduction. The change in Mn concentration could activate different groups of TPS, altering the composition of terpenes. In the current experiment, Mn significantly affected the concentration of oleanolic acid in leaves. Interestingly, this was the major compound found in stems. Manganese treatments significantly affected the oleanolic acid to oleuropein ratio (data not shown). The concentration of oleanolic acid in stems at 0.2 µM Mn was twice as high as that of oleuropein. While the level of oleuropein significantly increased at 24 µM Mn as compared to the 0.2 µM Mn and 12 µM Mn treatments, the concentration of oleanolic acid remained unchanged and, consequently, their ratio declined to 1.2 under 24 µM Mn treatment. In roots, oleanolic acid was undetected. A similar pattern was reported by Jimenez-Herrera et al. [61] for well-irrigated olive plants where oleuropein was the predominant biophenolic compound found in leaves and roots, while oleanolic acid dominated in stems. 

Among all of the investigated flavonoids, luteolin-7-*O*-glucoside was predominant in all plant parts. A strongly positive correlation between this flavonoid and Mn concentration was obtained with *r* = 0.81 and *p* = 0.01 (Table 2), which was in agreement with our previously reported results [55]. The significant increase in the concentration of luteolin-7-*O*-glucoside in leaves under the enhanced Mn supply (Figure 1) might imply its more specific functional aspect.

In order to modulate and control the synthesis of biophenolic compounds through plant nutrition, further investigations are required. Hopefully, such investigations will elucidate the intricate interplay of these plant secondary metabolites when exposed to excessive Mn concentrations.

## 4. Materials and Methods

### 4.1. Experimental Conditions, Treatments and Sampling

The experiment was conducted in a greenhouse at the Institute of Agriculture and Tourism in Poreč, (Latitude: 45°22′29.87″ N; Longitude: 13°60′31.60″), Croatia, from mid-February to mid-July under natural light, temperature, and photoperiod conditions. The average day/night temperature range varied between 10 and 35 °C.

One-year-old and self-rooted plantlets of the olive cultivar ‘Istarska bjelica’ were trained to one shoot, and grown in 3.5 L pots containing a substrate composed of perlite (Agroperl; Europerl d.o.o., Samobor, Croatia) and sand (Fuga Sand; Kema d.o.o., Puconci, Slovenia) blended in an equal ratio (*w*/*w*) [62]. The experiment was set up as a random block design with four repetitions. Each of three fertilisation treatments was represented by five plantlets per repetition. The total number of plantlets was 60.

For each fertilisation treatment, the full-strength Hoagland’s nutrient solution [7] was modified with different levels of Mn concentrations as follows: 0.2 µM Mn (0.2 μM MnSO_4_, to induce Mn deficiency), 12 µM Mn (12.0 μM MnSO_4_, to promote normal growth), and 24 µM Mn (24 μM MnSO_4_, to promote normal growth and ensure measurable differences in phenolic response in respect to 12 µM Mn). The selection of the concentrations was based on the results of preliminary experiments, carried out on the same cultivar under similar experimental conditions. The pH of the prepared solutions was corrected using 0.1 M H_2_SO_4_ and then measured using a Seven2Go pH meter (Mettler-Toledo GmbH, Gießen, Germany). The obtained values fluctuated from 5.99 to 6.13. Electric conductivity (EC) of the same solution was measured on the FG3 FiveGO conductometer (Mettler-Toledo GmbH, Gießen, Germany) and ranged from 1.57 (0.2 µM Mn) to 1.59 (24 µM Mn) dSm. The fertilisation treatments were applied twice a week, whereas the fertilisation dose was adjusted according to the plant growth requirements [63]. Nutrient solutions were renewed on a biweekly basis. Overaccumulation of salts in the substrate was avoided by applying 500 mL of tap water once per month [64].

At the beginning and the end of the experimental growth, different morphological parameters were measured: number of nodes, unfolded leaves (all leaves larger than 2 cm), and shoot length (cm).

Simultaneously, whole plants were sampled and divided into separate portions of leaves, stems, and roots. Samples were taken to the laboratory and rinsed twice with tap water, followed by 1% acetic acid, and finally double-distilled water. After the experiment’s completion, the total length (cm), area (cm^2^), diameter (mm), and volume (cm^3^) of previously cleaned roots were measured. Briefly, finely cut roots were placed in a plexiglas vessel (200 × 300 mm), positioned on an Epson Perfection V700 scanner (Seiko Epson Corporation, Nagano, Japan), and filled with deionised water up to 500 mL. Thereafter, root samples were arranged in the vessel to exclude overlapping and measured using a WinRHIZO^TM^ image analysis system (Re-gent Instruments Inc., Ottawa, Canada) [63]. Olive roots, stems, and leaves were then dried in an oven (Memmert Universal Oven UF160; Memmert GmbH+Co. KG, Schwabach, Germany) at 35 °C up to constant mass. After measuring the dry weight (DW), all samples were milled to a fine powder (0.2 mm sieves, Ultra Centrifugal mill ZM 200; Retsch Maschinen GmbH, Setzingen, Germany) and used for further analyses.

### 4.2. Chemicals

Acetonitrile (AcN), methanol (MeOH), and Folin–Ciocalteu reagent were purchased from Merck (Darmstadt, Germany), phosphoric acid from Sigma-Aldrich (St. Louis, MO, USA), and acetic acid, hydrogen peroxide, and nitric acid from VWR International S.A.S. (Fontenay-sous-Bois, France). Apigenin, apigenin-7-*O*-glucoside, catechin, hydroxytyrosol, luteolin, luteolin-7-*O*-glucoside, oleuropein, rutin, tyrosol, and verbascoside were purchased from Extrasynthese (Genay, France). All chemicals were of analytical grade purity and were used without further purification.

Deionised water was obtained using a Hydrolab 10 SP purification system (Hydrolab, Straszyn, Poland).

### 4.3. Sample Preparation

Biophenols were extracted as previously described [64] with some minor modifications. Oven-dried and finely ground olive roots (750 mg), stems (500 mg), and leaves (500 mg) were separately extracted with 20 mL of methanol 80% (*v*/*v*) in an ultrasonic bath (Sonorex Digitec; Bandelin electronic, Berlin, Germany) for 20 min. An aliquot (14 mL) of the extract was centrifuged for 7 min at 4000 rpm (Domel Centric 350; Železniki, Slovenia), and the supernatant was filtered through a 0.45 µm-pore cellulose acetate syringe filter.

### 4.4. Determination of TPC

TPC was determined using the colourimetric Folin–Ciocalteu method as described elsewhere [65]. Briefly, 250 μL of the extract was mixed with 15 mL of deionised water and 1.25 mL of Folin–Ciocalteu reagent in a 25 mL flask. After 3 min, 2.5 mL of a saturated sodium carbonate solution was added, and the flask was refilled with deionised water to the mark. The obtained mixture was left to stand for 90 min at room temperature in a dark place before the absorbance at 725 nm was measured (Perkin-Elmer UV/VIS Lambda Bio 40 spectrophotometer; PerkinElmer Instruments, Waltham, MA, USA). The final results were calculated against a standard curve of pure caffeic acid (Sigma-Aldrich, Steineheim, Germany), with a concentration range from 0.0256 to 1.0 mg/mL (R^2^ = 0.9998), and expressed as mg of caffeic acid equivalent per 100 g of dry matter. All measurements were carried out in triplicate.

### 4.5. High-Performance Liquid Chromatography (HPLC)

HPLC analysis was performed on a Thermo Ultimate 3000 System, equipped with a degasser, a binary pump, an autosampler, a column oven, and a UV/Vis detector that was capable of simultaneously measuring four different wavelengths (ThermoFisher Scientific, Waltham, MA, USA). The separation of biophenols was performed using a Lichrospher 100 RP-18 (250 mm × 4 mm, 5 µm) analytical column with a Lichrospher 100 pre-column (4 mm × 4 mm, 5 µm), both supplied by Agilent Technologies (Santa Clara, CA, USA), and the column temperature was kept constant at 25 °C. For the chromatographic separation, 0.2% phosphoric acid in water was used as solvent A, and MeOH: ACN (1:1) as solvent B. The elution was performed at a flow rate of 0.8 mL/min, and the gradient is described below: 10% B for 0.5 min, then from 10–16.5% B in 24.5 min to 30% B in 55 min and finally to 100% B in 15 min, keeping it constant for 5 min. The wavelength was set at 250 nm for luteolin-7-*O*-glucoside and oleuropein, 280 nm for apigenin-7-*O*-glucoside, catechin, hydroxytyrosol, and tyrosol, 305 nm for apigenin, caffeic acid and verbascoside, and 370 nm for luteolin and rutin. Identification was performed by comparing retention times of the target compounds in the sample extracts with those of pure standards. Quantification was conducted using an external standard method. The calibration curves for individual biophenols were obtained using five calibration levels made by appropriate dilutions of the stock standard solutions and calibration curves, with R^2^ ≥ 0.999 accepted for concentration calculation. 

### 4.6. Manganese Concentration Measurement

A mass of 200 mg of dried sample was accurately weighed into a microwave pressure vessel. After adding 6 mL of concentrated nitric acid and 2 mL of 30% hydrogen peroxide, samples were digested using a microwave system (Milestone ETHOS UP; Sorisole, Italy) over 40 min at 1800 W and 200 °C. After cooling, the solutions were diluted to 25 mL with deionised water and quantitatively transferred to the appropriate vials. One replication per digestion method was performed for each sample. Samples were then analysed by an inductively coupled plasma atomic emission (ICP-AE) spectrometer (ICPE-9820; Shimadzu, Kyoto, Japan), equipped with an autosampler (AS-10; Shimadzu, Kyoto, Japan). 

Single element standard solution of Mn (Inorganic Ventures, Christiansburg, VA, USA) was used to control the plasma positioning and preparation of calibration standard solutions. The calibration standard was prepared by serial dilution of a stock solution (concentration range from 0.01 to 15 mg/L). The accuracy of a procedure was tested by certified reference materials (WEPAL, Wageningen, The Netherlands) prepared in the same way as samples. The most suitable emission lines without background and spectral interferences were selected for the detection.

### 4.7. Concentration to Quantity Conversion

The quantities of Mn and bioactive compounds in each tissue were calculated using the following formula:

$$\;\mathrm{Quantity}\;{\left(\mathrm{Mn}\;\mathrm{or}\;\mathrm{bioactive}\;\mathrm{compound}\right)}_{\mathrm{tissue}}=\;\mathrm{Concentration}\;{\left(\mathrm{Mn}\;\mathrm{or}\;\mathrm{bioactive}\;\mathrm{compound}\right)}_{\mathrm{tissue}\;}\times {\;\mathrm{DW}}_{\mathrm{tissue}}$$

where the concentration is the result obtained from TPC, HPLC or ICP analysis and DW is the mass of the oven-dried tissue/plant in question. 

### 4.8. TF, MnUE, Mn Distribution

The Mn TF was calculated as the ratio of the quantity of Mn in the leaves or stems and the quantity of Mn in the roots [66].

MnUE was calculated as the ratio of the plant DW and the total quantity of Mn [46].

The percent Mn distribution was calculated as the ratio of the quantity of Mn in the leaves, stems or roots and the total quantity of Mn [46].

### 4.9. Statistical Analysis

As mentioned before, the experiment was set up as a random block design in four replications. Analysis of variance (ANOVA) was performed for all the data. Multiple comparisons of means were based on Tukey’s test at *p* ≤ 0.05. Correlation coefficients were determined between Mn quantity, total biophenols and individual biophenolic compounds. Statistical analysis was performed using Statistica 13.2 software (StatSoft^®^, Palo Alto, CA, USA).

## 5. Conclusions

To conclude, Mn was mainly retained in the roots of ‘Istarska bjelica’ olive plantlets that were cultivated hydroponically, and only its smaller portion was translocated to leaves and shoots. Thus, although the vegetative growth of the upper plant parts was not affected by differences in Mn supply, the root elongation, root area, and volume were the highest at 12 µM Mn, thus confirming this concentration as optimal dose of Mn for root development of the ‘Istarska bjelica’ cultivar. A positive correlation of the total per plant quantity of oleuropein, luteolin-7-*O*-glucoside, hydroxytyrosol, caffeic acid, vanillic acid, rutin, and luteolin with the degree of Mn fertilisation was observed. Oleuropein was confirmed to be the most abundant biophenolic compound in leaves and its concentration in leaves and stems increased under manganese supply. The concentration of the most important flavonoid, luteolin-7-*O*-glucoside, increased in all tissues under the application of the highest Mn concentration (24 µM Mn). Oleanolic acid was a predominant compound in stems, while in roots it was not detected. Generally, Mn nutrition increased concentrations of individual biophenols but the effect was dependent on the compound and tissue in question. Our observations could help to better understand the distribution and translocation of valuable bioactive compounds in different olive tissues under Mn nutrition. Still, there are many unresolved issues and further studies need to be conducted to elucidate the relationship between Mn fertilisation and olive secondary metabolism. 

## Figures and Tables

**Table 1:** Root morphological parameters of ‘Istarska bjelica’ olive cv. treated with three different manganese (Mn) concentrations.

Source of Variation	Total Length (cm/plant)	Surface Area (cm^2^/plant)	Diameter (mm/plant)	Volume (cm^3^/plant)
Treatment				
0.2 µM Mn	2020.03 ± 142.60 ^b^	410.91 ± 24.18 ^b^	0.65 ± 0.01	6.67 ± 0.32 ^b^
12 µM Mn	2444.86 ± 62.72 ^a^	505.71 ± 15.93 ^a^	0.66 ± 0.02	8.35 ± 0.44 ^a^
24 µM Mn	1954.38 ± 25.86 ^b^	392.32 ± 7.61 ^b^	0.63 ± 0.01	6.28 ± 0.17 ^b^
*p*-value	*	**	n.s.	*

Results are expressed as means ± standard errors (*n* = 4). Different superscript lowercase letters in a column represent statistically significant differences between mean values for each main effect at *p* < 0.05 obtained by ANOVA main effects and Tukey’s test. Significance: ***—*p* < 0.001; **—*p* < 0.01; *—*p* < 0.05.

**Table 2:** Correlation coefficients between the total per plant quantity of Mn and simple biophenols, phenolic acids, and flavonoids of ‘Istarska bjelica’ olive cv. treated with three different manganese (Mn) concentrations.

	Simple Biophenols			Phenolic Acids			Triterpene	Secoiridoid	Flavonoids						TPQ
	HTYR	TYR	Vanillin	VERB	Vanillic acid	Caffeic acid	Oleanolic acid	Oleuropein	Catechin	Luteolin	Apigenin	Apigenin-*O*	Rutin	Luteolin-*O*	
r	0.61	0.14	−0.26	−0.20	0.62	0.89	0.36	0.83	0.57	−0.70	0.18	−0.30	0.75	0.81	0.65
*p*	0.036	0.663	0.420	0.540	0.031	0.000	0.251	0.001	0.053	0.012	0.345	0.570	0.005	0.001	0.022

HTYR—hydroxytyrosol; TYR—tyrosol; VERB—verbascoside; Apigenin-*O*—apigenin-7-*O*-glucoside; Luteolin-*O*—luteolin-7-*O*-glucoside; TPQ—total phenolic quantity. Relationships among the observed variables are expressed as correlation coefficients (*r*) and significance (*p*), (*n* = 12).

**Table 3:** Distribution (%) of the total per plant quantity of manganese (Mn) in leaves, stems, and roots of ‘Istarska bjelica’ olive cv., translocation factor (TF), and manganese use efficiency (MnUE) values.

Source of Variation	Distribution (%)			TF		MnUE
	Leaves	Stems	Roots	Leaves	Stems	(mg of the Total Plant DW/µg of the Total per Plant Quantity of Mn)
Treatment						
0.2 µM Mn	27.7 ± 0.05 ^a^	11.0 ± 0.01 ^a^	61.3 ± 0.05 ^b^	0.48 ± 0.12 ^a^	0.18 ± 0.03 ^a^	82.77 ± 0.01 ^a^
12 µM Mn	17.8 ± 0.01 ^ab^	6.2 ± 0.01 ^b^	76.0 ± 0.02 ^ab^	0.24 ± 0.02 ^ab^	0.08 ± 0.01 ^b^	22.15 ± 0.00 ^b^
24 µM Mn	12.6 ± 0.01 ^b^	4.2 ± 0.00 ^b^	83.1 ± 0.01 ^a^	0.15 ± 0.02 ^b^	0.05 ± 0.00 ^b^	12.71 ± 0.00 ^b^
*p*-value	*	**	*	*	**	**

Results are expressed as means ± standard errors (*n* = 4). Different superscript lowercase letters in a column represent statistically significant differences between mean values for each main effect at *p* < 0.05 obtained by ANOVA main effects and Tukey’s test. Significance: ***—*p* < 0.001; **—*p* < 0.01; *—*p* < 0.05. DW—dry weight.

## Data Availability

The data presented in this study are available on request from the corresponding author.

## Supplementary Materials

The following are available online at https://www.mdpi.com/article/10.3390/plants10081724/s1, Figure S1: Concentration of simple biophenols, phenolic acids, flavonoids and total biophenols in leaves of ‘Istarska bjelica’ olive cv. treated with three different manganese (Mn) concentrations. Table S2: Concentration of simple biophenols, phenolic acids, flavonoids, and total biophenols in the stem of ‘Istarska bjelica’ olive cv. treated with three different manganese (Mn) concentrations. Table S3: Concentration of simple biophenols, phenolic acids, flavonoids, and total biophenols in the root of ‘Istarska bjelica’ olive cv. treated with three different manganese (Mn) concentrations. Table S4: The number of nodes and leaves, the plant height, the root, stem, and leaves DW of ‘Istarska bjelica’ olive cv. treated with three different manganese (Mn) concentrations. Table S5: The total per plant quantity of simple biophenols, phenolic acids and flavonoids of ‘Istarska bjelica’ olive cv. treated with three different manganese (Mn) concentrations.

## References

[1] Zarrouk W., Baccouri B., Taamalli W., Trigui A., Daoud D., Zarrouk M. (2009). Oil fatty acid composition of eighteen Mediterranean olive varieties cultivated under the arid conditions of Boughrara (southern Tunisia). Grasas Y Aceites.

[2] Gómez-Rico A., Salvador M.D., Fregapane G. (2009). Virgin olive oil and olive fruit minor constituents as affected by irrigation management based on SWP and TDF as compared to ETc in medium-density young olive orchards (*Olea europaea* L. cv. Cornicabra and Morisca). Food Res. Int..

[3] Talhaoui N., Taamalli A., Gómez-Caravaca A.M., Fernández-Gutiérrez A., Segura-Carretero A. (2015). Phenolic compounds in olive leaves: Analytical determination, biotic and abiotic influence, and health benefits. Food Res. Int..

[4] Lama-Muñoz A., del Mar Contreras M., Espínola F., Moya M., de Torres A., Romero I., Castro E. (2019). Extraction of oleuropein and luteolin-7-O-glucoside from olive leaves: Optimization of technique and operating conditions. Food Chem..

[5] Xie P.J., Huang L.X., Zhang C.H., You F., Wang C.Z., Zhou H. (2015). Reduced-pressure boiling extraction of oleuropein coupled with ultrasonication from olive leaves (*Olea europaea* L.). Adv. Mater. Sci. Eng..

[6] Žuntar I., Putnik P., Kovačević B.D., Nutrizio M., Šupljika F., Poljanec A., Dubrović I., Barba F.J., Jambrak A.R. (2019). Phenolic and Antioxidant Analysis of Olive Leaves. Foods.

[7] Talhaoui N., Vezza T., Gómez-Caravaca A.M., Fernández-Gutiérrez A., Gálvez J., Segura-Carretero A. (2016). Phenolic compounds and in vitro immunomodulatory properties of three Andalusian olive leaf extracts. J. Funct. Foods.

[8] Hong Y.H., Song C., Shin K.K., Choi E., Hwang S.H., Jang Y.J., Taamalli A., Yum J., Kim J.H., Kim E. (2021). Tunisian *Olea europaea* L. leaf extract suppresses Freund’s complete adjuvant-induced rheumatoid arthritis and lipopolysaccharide-induced inflammatory responses. J. Ethnopharmacol..

[9] Thielmann J., Kohnen S., Hauser C. (2017). Antimicrobial activity of *Olea europaea* Linné extracts and their applicability as natural food preservative agents. Int. J. Food Microbiol..

[10] Lorzadeh N., Kazemirad Y., Kazemirad N. (2021). Treatment of genital herpes using olive leaf extract. Clin. Case Rep..

[11] Taamalli A., Arráez Román D., Gómez Caravaca A.M., Zarrouk M., Segura Carretero A. (2018). Geographical Characterization of Tunisian Olive Tree Leaves (cv. Chemlali) Using HPLC-ESI-TOF and IT/MS Fingerprinting with Hierarchical Cluster Analysis. J. Anal. Methods Chem..

[12] Andrikopoulos N.K., Antonopoulou S., Kaliora A.C. (2002). Oleuropein inhibits LDL oxidation induced by cooking oil frying by-products and platelet aggregation induced by platelet-activating factor. LWT Food Sci. Technol..

[13] Kashaninejad M., Sanz M.T., Blanco B., Beltrán S., Niknam S.M. (2020). Freeze dried extract from olive leaves: Valorisation, extraction kinetics and extract characterization. Food Bioprod. Process..

[14] Kritikou E., Kalogiouri N.P., Kolyvira L., Thomaidis N.S. (2020). Target and Suspect HRMS Metabolomics for the Determination of Functional Ingredients in 13 Varieties of Olive Leaves and Drupes from Greece. Molecules.

[15] Mechri B., Tekaya M., Hammami M., Chehab H. (2020). Effects of drought stress on phenolic accumulation in greenhouse-grown olive trees (*Olea europaea*). Biochem. Syst. Ecol..

[16] Ammar S., del Contreras M.M., Gargouri B., Segura-Carretero A., Bouaziz M. (2017). RP-HPLC-DAD-ESI-QTOF-MS based metabolic profiling of the potential *Olea europaea* by-product “wood” and its comparison with leaf counterpart. Phytochem. Anal..

[17] Altarejos J., Salido S., Pérez-Bonilla M., Linares-Palomino P.J., Van Beek T.A., Nogueras M., Sánchez A. (2005). Preliminary assay on the radical scavenging activity of olive wood extracts. Fitoterapia.

[18] Pérez-Bonilla M., Salido S., Van Beek T.A., Altarejos J. (2014). Radical-scavenging compounds from olive tree (*Olea europaea* L.) wood. J. Agric. Food Chem..

[19] Entus R., Poling M., Herrmann K.M. (2015). Redox Regulation of Arabidopsis 3-Deoxy-D-arabino-Heptulosonate 7-Phosphate Synthase. Plant Physiol..

[20] Alejandro S., Höller S., Meier B., Peiter E. (2020). Manganese in Plants: From Acquisition to Subcellular Allocation. Front. Plant Sci..

[21] Brown P.H., Graham R.D., Nicholas D.J.D. (1984). The effects of manganese and nitrate supply on the levels of phenolics and lignin in young wheat plants. Plant Soil.

[22] Muzolf-Panek M., Kleiber T., Kaczmarek A. (2017). Effect of increasing manganese concentration in nutrient solution on the antioxidant activity, vitamin C, lycopene and polyphenol contents of tomato fruit. Food Addit. Contam. Part A Chem. Anal. Control. Expo. Risk Assess..

[23] Nazari M., Zarinkamar F., Niknam V. (2018). Changes in primary and secondary metabolites of Mentha aquatica L. exposed to different concentrations of manganese. Environ. Sci. Pollut. Res..

[24] Farzadfar S., Zarinkamar F., Hojati M. (2017). Magnesium and manganese affect photosynthesis, essential oil composition and phenolic compounds of Tanacetum parthenium. Plant Physiol. Biochem..

[25] Pasković I., Herak Ćustić M., Pecina M., Bronić J., Ban D., Radić T., Pošćić F., Jukić Špika M., Soldo B., Palčić I. (2019). Manganese soil and foliar fertilization of olive plantlets: The effect on leaf mineral and phenolic content and root mycorrhizal colonization. J. Sci. Food Agric..

[26] Chatzistathis T., Therios I., Alifragis D. (2009). Differential uptake, distribution within tissues, and use efficiency of Manganese, Iron, and Zinc by olive cultivars Kothreiki and Koroneiki. HortScience.

[27] Millaleo R., Reyes-Díaz M., Ivanov A.G., Mora M.L., Alberdi M. (2010). Manganese as essential and toxic element for plants: Transport, accumulation and resistance mechanisms. J. Soil Sci. Plant Nutr..

[28] Ma G., Li J., Li J., Li Y., Gu D., Chen C., Cui J., Chen X. (2018). Plant Science OsMTP11, a trans-Golgi network localized transporter, is involved in manganese tolerance in rice. Plant Sci..

[29] Zhang C., Liu P. (2017). The lipid droplet: A conserved cellular organelle. Protein Cell.

[30] Yamada K., Nagano A.J., Nishina M., Hara-nishimura I., Nishimura M. (2013). Identification of two novel endoplasmic reticulum body-specific integral membrane proteins. Plant Physiol..

[31] Schneider A.C., Chun H., Tefanovic S., Baldwin B.G. (2018). Punctuated plastome reduction and host-parasite horizontal gene transfer in the holoparasitic plant genus Aphyllon. Proc. R. Soc. B Biol. Sci..

[32] Frank J., Happeck R., Meier B., Hoang M.T.T., Stribny J., Hause G., Ding H., Morsomme P., Baginsky S., Peiter E. (2019). Chloroplast-localized BICAT proteins shape stromal calcium signals and are required for efficient photosynthesis. New Phytol..

[33] Corpas F.J., Barroso J.B., Palma J.M., Rodriguez-Ruiz M. (2017). Plant peroxisomes: A nitro-oxidative cocktail. Redox Biol..

[34] Eroglu S., Giehl R.F.H., Meier B., Takahashi M., Terada Y., Ignatyev K., Andresen E., Küpper H., Peiter E., von Wirén N. (2017). Metal tolerance protein 8 mediates manganese homeostasis and iron reallocation during seed development and germination. Plant Physiol..

[35] Lanquar V., Ramos M.S., Lelièvre F., Barbier-Brygoo H., Krieger-Liszkay A., Krämer U., Thomine S. (2010). Export of vacuolar manganese by AtNRAMP3 and AtNRAMP4 is required for optimal photosynthesis and growth under manganese deficiency. Plant Physiol..

[36] Connor D.J., Fereres E. (2010). The Physiology of Adaptation and Yield Expression in Olive. Hortic. Rev. (Am. Soc. Hortic. Sci)..

[37] Zhang T., Li R., Xing J., Yan L., Wang R., Zhao Y. (2018). The YUCCA-auxin-wox11 module controls crown root development in rice. Front. Plant Sci..

[38] Tracy J.E. (1991). Manganese Toxicity in Avocado (*Persea americana* Mill.). Calif. Avocado Soc..

[39] Alam S., Kamei S., Kawai S. (2001). Amelioration of manganese toxicity in barley with iron. J. Plant Nutr..

[40] Quartin V.M.L., Antunes M.L., Muralha M.C., Sousa M.M., Nunes M.A. (2001). Mineral imbalance due to manganese excess in triticales. J. Plant Nutr..

[41] Sarkar D., Pandey S.K., Sud K.C., Chanemougasoundharam A. (2004). In vitro characterization of manganese toxicity in relation to phosphorus nutrition in potato (*Solanum tuberosum* L.). Plant Sci..

[42] Chatzistathis T.A., Papadakis I.E., Therios I.N., Giannakoula A., Dimassi K. (2011). Is chlorophyll fluorescence technique a useful tool to assess manganese deficiency and toxicity stress in olive plants?. J. Plant Nutr..

[43] Page V., Feller U. (2015). Heavy metals in crop plants: Transport and redistribution processes on the whole plant level. Agronomy.

[44] Verlag F., Lidon F.C. (2001). Tolerance of rice to excess manganese in the early stages of vegetative growth. Characterisation Manganese Accumul..

[45] Andrew B.C.S., Hegarty M.P. (1969). Comparative responses to manganese excess of eight tropical and four temperate pasture legume species. Aust. J. Agric. Res..

[46] Chatzistathis T., Papadakis I., Therios I., Patakas A., Giannakoula A., Menexes G. (2012). Differential Response of Two Olive Cultivars To Excess Manganese. J. Plant Nutr..

[47] Rengel Z., Graham R.D., Pedler J.F. (1993). Manganese nutrition and accumulation of phenolics and lignin as related to differential resistance of wheat genotypes to the take-all fungus. Plant Soil.

[48] Marchiosi R., dos Santos W.D., Constantin R.P., de Lima R.B., Soares A.R., Finger-Teixeira A., Mota T.R., de Oliveira D.M., de Paiva Foletto-Felipe M., Abrahão J. (2020). Biosynthesis and Metabolic Actions of Simple Phenolic Acids in Plants. Phytochem. Rev..

[49] Leopoldini M., Chiodo S.G., Russo N., Toscano M. (2011). Detailed investigation of the OH radical quenching by natural antioxidant caffeic acid studied by quantum mechanical models. J. Chem. Theory Comput..

[50] Cardinali A., Pati S., Minervini F., D’Antuono I., Linsalata V., Lattanzio V. (2012). Verbascoside, isoverbascoside, and their derivatives recovered from olive mill wastewater as possible food antioxidants. J. Agric. Food Chem..

[51] Mechri B., Tekaya M., Hammami M., Chehab H. (2019). Root verbascoside and oleuropein are potential indicators of drought resistance in olive trees (*Olea europaea* L.). Plant Physiol. Biochem..

[52] Obied H.K., Prenzler P.D., Ryan D., Servili M., Taticchi A., Esposto S., Robards K. (2008). Biosynthesis and biotransformations of phenol-conjugated oleosidic secoiridoids from *Olea europaea* L.. Nat. Prod. Rep..

[53] Ryan D., Robards K. (1998). Phenolic compounds in olives. Analyst.

[54] Alagna F., Geu-Flores F., Kries H., Panara F., Baldoni L., O’Connor S.E., Osbourn A. (2016). Identification and characterization of the iridoid synthase involved in oleuropein biosynthesis in olive (*Olea europaea*) fruits. J. Biol. Chem..

[55] Pasković I., Lukić I., Žurga P., Germek V.M., Brkljača M., Koprivnjak O., Major N., Grozić K., Franić M., Ban D. (2020). Temporal variation of phenolic and mineral composition in olive leaves is cultivar dependent. Plants.

[56] Lukić I., Pasković I., Žurga P., Germek V.M., Brkljača M., Marcelić Š., Ban D., Grozić K., Lukić M., Užila Z. (2020). Determination of the variability of biophenols and mineral nutrients in olive leaves with respect to cultivar, collection period and geographical location for their targeted and well-timed exploitation. Plants.

[57] Garcı P. (1996). Effect of Metal Cations on the Chemical Oxidation of Olive o-Diphenols in Model Systems. J. Agric. Food Chem..

[58] Jäger S., Trojan H., Kopp T., Laszczyk M.N., Scheffler A. (2009). Pentacyclic triterpene distribution in various plants—Rich sources for a new group of multi-potent plant extracts. Molecules.

[59] Ghannadnia M., Haddad R., Zarinkamar F., Sharifi M. (2014). Manganese treatment effects on terpene compounds of Cuminum cyminum flowers. Ind. Crop. Prod..

[60] de Alvarenga E.S., Silva S.A., Barosa L.C.A., Demuner A.J., Parreira A.G., Ribeiro R.I.M.A., Marcussi S., Ferreira J.M.S., Resende R.R., Granjeiro P.A. (2011). synthesis and evaluation of sesquiterpene lactone inhibitors of phospholipase A2 from Bothrops jararacussu. Toxicon.

[61] Jiménez-Herrera R., Pacheco-López B., Peragón J. (2019). Water stress, irrigation and concentrations of pentacyclic triterpenes and phenols in *Olea europaea* L. Cv. picual olive trees. Antioxidants.

[62] Perica S., Goreta S., Selak G.V. (2008). Growth, biomass allocation and leaf ion concentration of seven olive (*Olea europaea* L.) cultivars under increased salinity. Sci. Hortic..

[63] Pasković I., Pecina M., Bronić J., Perica S., Ban D., Ban S.G., Pošćić F., Palčić I., Herak Ćustić M. (2018). Synthetic Zeolite A as Zinc and Manganese Fertilizer in Calcareous Soil. Commun. Soil Sci. Plant Anal..

[64] Popović M., Jukić Špika M., Veršić Bratinčević M., Ninčević T., Matešković A., Mandušić M., Rošin J., Nazalić M., Dunkić V., Vitanović E. (2021). Essential Oil Volatile Fingerprint Differentiates Croatian cv. Oblica from Other *Olea europaea* L. Cultivars. Molecules.

[65] Pasković I., Soldo B., Talhaoui N., Palčić I., Brkljača M., Koprivnjak O., Majetić Germek V., Ban D., Klanjac J., Franić M. (2019). Boron foliar application enhances oleuropein level and modulates volatile compound composition in olive leaves. Sci. Hortic..

[66] Zvobgo G., Lwalaba J.L.W., Sehar S., Mapodzeke J.M., Shamsi I.H., Zhang G. (2018). The Tolerance Index and Translocation Factor Were Used to Identify the Barley Genotypes with High Arsenic Stress Tolerance. Commun. Soil Sci. Plant Anal..

