# Supplementary material for: Biophenolic Profile Modulations in Olive Tissues as Affected by Manganese Nutrition
Source: Plants (Basel). 2021 Aug 20;10(8):1724. doi: 10.3390/plants10081724 (PMC8402200; doi:10.3390/plants10081724)
Supplement: Supplementary file 1 [file plants-10-01724-s001.zip › plants-1306178-SI.pdf]

Table S1. Concentration of simple biophenols, phenolic acids, flavonoids and total biophenols in leaves of 'Istarska bjelica' olive cv. treated with three different manganese (Mn) concentrations.

**Source of variation**	**Simple biophenols**			**Phenolic acid**	**Flavonoids**					**Total biophenols**
	**Hydroxy-tyrosol**	**Tyrosol**	**Vanillin**	**Vanillic acid**	**Catechin**	**Luteolin**	**Apigenin**	**Apigenin-7-*O*-glucoside**	**Rutin**	
	**(mg/100 g DW)**									
**Treatment**										
0.2 μM Mn	35.29 ± 2.83	27.32 ± 4.47	0.52 ± 0.07	1.42 ± 0.14	26.76 ± 1.55	32.45 ± 3.31^{a}	2.94 ± 0.17^{a}	10.23 ± 1.50	52.73 ± 5.73	5150.56 ± 162.66
12 μM Mn	34.00 ± 1.61	26.75 ± 2.44	0.63 ± 0.02	1.43 ± 0.19	26.85 ± 0.97	24.38 ± 3.86^{a}	2.75 ± 0.31^{a}	11.70 ± 0.29	56.25 ± 7.02	5537.62 ± 228.43
24 μM Mn	38.69 ± 3.18	25.4 ± 3.06	0.56 ± 0.00	1.09 ± 0.28	28.07 ± 0.65	13.31 ± 1.35^{b}	1.57 ± 0.14^{b}	10.64 ± 0.57	67.88 ± 4.97	6095.81 ± 194.72
***p*-value**	n.s.	n.s.	n.s.	n.s.	**n.s.**	**	*	**n.s.**	**n.s.**	**n.s.**

Results are expressed as means ± standard errors (n=4). Different superscript lowercase letters in the column represent statistically significant differences between mean values for each main effect at $p < 0.05$ obtained by ANOVA main effects and Tukey's test. Significance: ***—$p < 0.001$. **—$p < 0.01$. *—$p < 0.05$. DW — dry weight.

Table S2. Concentration of simple biophenols, phenolic acids, flavonoids, and total biophenols in stems of 'Istarska bjelica' olive cv. treated with three different manganese (Mn) concentrations.

**Source of variation**	**Simple biophenols**			**Phenolic acids**		**Flavonoids**			**Total biophenols**
	**Hydroxytyrosol**	**Tyrosol**	**Vanillin**	**Vanillic acid**	**Caffeic acid**	**Luteolin**	**Apigenin**	**Rutin**	
	**(mg/100 g DW)**								
**Treatment**									
0.2 μM Mn	16.93 ± 0.4^{b}	9.93 ± 0.85	1.54 ± 0.05^{b}	9.48 ± 0.8^{b}	2.35 ± 0.17^{b}	3.38 ± 0.27	1.69 ± 0.17^{b}	7.57 ± 1.15^{b}	1434.37 ± 82.13
12 μM Mn	17.91 ± 1.15^{b}	11.42 ± 0.42	1.68 ± 0.11^{b}	11.31 ± 1.17^{ab}	2.66 ± 0.13^{b}	3.36 ± 0.21	1.78 ± 0.14^{b}	9.81 ± 0.91^{b}	1677.80 ± 89.36
24 μM Mn	23.56 ± 0.68^{a}	16.49 ± 2.56	2.23 ± 0.06^{a}	14.82 ± 0.89^{a}	3.93 ± 0.37^{a}	3.92 ± 0.05	2.43 ± 0.24^{a}	14.11 ± 0.31^{a}	2241.52 ± 55.30
***p*-value**	**	**n.s.**	**	*	*	**n.s.**	*	**	**n.s.**

Results are expressed as means ± standard errors (n=4). Different superscript lowercase letters in the column represent statistically significant differences between mean values for each main effect at $p < 0.05$ obtained by ANOVA main effects and Tukey's test. Significance: ***—$p < 0.001$. **—$p < 0.01$. *—$p < 0.05$. DW — dry weight.

Table S3. Concentration of simple biophenols, phenolic acids, flavonoids, and total biophenols in roots of 'Istarska bjelica' olive cv. treated with three different manganese (Mn) concentrations.

**Source of variation**	**Simple biophenols**			**Phenolic acids**		**Flavonoids**	**Total biophenols**
	**Hydroxytyrosol**	**Tyrosol**	**Vanillin**	**Vanillic acid**	**Caffeic acid**	**Apigenin-7-*O*-glucoside**	
	**(mg/100 g DW)**						
**Treatment**							
0.2 μM Mn	5.73 ± 0.57	16.81 ± 1.24[a]	9.57 ± 1.24	7.32 ± 0.43	0.46 ± 0.06[a]	0.70 ± 0.08[b]	2226.68 ± 282.20
12 μM Mn	4.78 ± 0.86	13.33 ± 1.76[b]	8.20 ± 1.91	7.01 ± 0.47	0.25 ± 0.03[b]	1.07 ± 0.31[ab]	1573.05 ± 266.61
24 μM Mn	5.17 ± 0.56	13.07 ± 0.62[b]	6.89 ± 1.41	6.92 ± 0.11	0.21 ± 0.03[b]	1.63 ± 0.13[a]	1556.50 ± 171.35
***p*-value**	**n.s.**	*	**n.s.**	**n.s.**	**	*	**n.s.**

Results are expressed as means ± standard errors (n=4). Different superscript lowercase letters in a column represent statistically significant differences between mean values for each main effect at $p < 0.05$ obtained by ANOVA main effects and Tukey's test. Significance: ***—$p < 0.001$. **—$p < 0.01$. *—$p < 0.05$. DW — dry weight.

Table S4. The number of nodes and leaves, the plant height, the root, stem, and leaves DW of 'Istarska bjelica' olive cv. treated with three different manganese (Mn) concentrations.

**Source of variation**	**Number of nodes**	**Number of leaves**	**Plant height (cm)**	**Root DW (g/plant)**	**Leaves DW (g/plant)**	**Stem DW (g/plant)**
**Treatment**						
0.2 μM Mn	20.00 ± 0.91	38.75 ± 1.38	67.68 ± 5.30	2.58 ± 0.20	8.40 ± 0.73	6.99 ± 0.67
12 μM Mn	21.00 ± 1.58	40.00 ± 4.81	73.20 ± 11.41	2.56 ± 0.05	9.11 ± 0.21	7.62 ± 0.20
24 μM Mn	19.50 ± 0.50	38.75 ± 1.25	70.25 ± 8.92	2.18 ± 0.10	7.79 ± 0.58	7.86 ± 0.36
***p*-value**	**n.s.**	**n.s.**	**n.s.**	**n.s.**	**n.s.**	**n.s.**

Results are expressed as means ± standard errors (n=4). Different superscript lowercase letters in a column represent statistically significant differences between mean values for each main effect at $p < 0.05$ obtained by ANOVA main effects and Tukey's test. Significance: ***—$p < 0.001$, **—$p < 0.01$, *—$p < 0.05$. DW—dry weight.

Table S5. The total per plant quantity of simple biophenols, phenolic acids and flavonoids of 'Istarska bjelica' olive cv. treated with three different manganese (Mn) concentrations.

Source of variation	Simple biophenols			Phenolic acids		Flavonoids				
	Hydroxytyrosol	Tyrosol	Vanillin	Vanillic acid	Caffeic acid	Catechin	Luteolin	Apigenin	Apigenin-7-*O*-glucoside	Rutin
	(mg/plant)									
**Treatment**										
0.2 μM Mn	4.01 ± 0.36	3.20 ± 0.56	0.41 ± 0.06	1.08 ± 0.09	0.26 ± 0.01 [b]	1.94 ± 0.16	2.62 ± 0.44	0.36 ± 0.01	0.81 ± 0.15	4.25 ± 0.34
12 μM Mn	4.33 ± 0.09	3.41 ± 0.13	0.41 ± 0.05	1.31 ± 0.08	0.31 ± 0.01[b]	2.13 ± 0.06	2.19 ± 0.31	0.39 ± 0.02	0.99 ± 0.05	5.20 ± 0.55
24 μM Mn	4.96 ± 0.20	3.59 ± 0.50	0.36 ± 0.03	1.38 ± 0.07	0.37 ± 0.02[a]	2.28 ± 0.06	1.38 ± 0.17	0.33 ± 0.03	0.94 ± 0.09	6.42 ± 0.33
***p*-value**	**n.s.**	**n.s.**	**n.s.**	**n.s.**	**	**n.s.**	**n.s.**	**n.s.**	**n.s.**	**n.s.**

Results are expressed as means ± standard errors. Different superscript lowercase letters in a column represent statistically significant differences between mean values for each main effect at $p < 0.05$ obtained by ANOVA main effects and Tukey's test. Significance: ***—$p < 0.001$. **—$p < 0.01$. *—$p < 0.05$.
